# Novel Wild-Type *Pediococcus* and *Lactiplantibacillus* Strains as Probiotic Candidates to Manage Obesity-Associated Insulin Resistance

**DOI:** 10.3390/microorganisms12020231

**Published:** 2024-01-23

**Authors:** Paraskevi Somalou, Eleftheria Ieronymaki, Kyriaki Feidaki, Ioanna Prapa, Electra Stylianopoulou, Katerina Spyridopoulou, George Skavdis, Maria E. Grigoriou, Panayiotis Panas, Anagnostis Argiriou, Christos Tsatsanis, Yiannis Kourkoutas

**Affiliations:** 1 Department of Molecular Biology & Genetics, Democritus University of Thrace, 68100 Alexandroupolis, Greece; swmalouparaskevi@gmail.com (P.S.); iprapa@mbg.duth.gr (I.P.); electrastyl@gmail.com (E.S.); aikspiridopoulou@gmail.com (K.S.); gskavdis@mbg.duth.gr (G.S.); mgrigor@mbg.duth.gr (M.E.G.); 2 Laboratory of Clinical Chemistry, Department of Laboratory Medicine, Medical School, University of Crete, 71003 Crete, Greece; eleftheriaier@hotmail.com (E.I.); tsatsani@uoc.gr (C.T.); 3 Institute of Applied Sciences, Centre for Research and Technology, 57001 Thessaloniki, Greece; riafeidaki@gmail.com (K.F.); argiriou@certh.gr (A.A.); 4 Department of Food Science and Nutrition, University of the Aegean, 81400 Lemnos, Greece; 5 QLC, 26442 Patras, Greece; panas@qlc.gr; 6 Institute for Molecular Biology and Biotechnology, FORTH, 71100 Heraklion, Greece

**Keywords:** probiotics, *Pediococcus*, *Lactiplantibacillus*, obesity, insulin resistance, type2 diabetes, high-fat diet

## Abstract

As the food and pharmaceutical industry is continuously seeking new probiotic strains with unique health properties, the aim of the present study was to determine the impact of short-term dietary intervention with novel wild-type strains, isolated from various sources, on high-fat diet (HFD)-induced insulin resistance. Initially, the strains were evaluated in vitro for their ability to survive in simulated gastrointestinal (GI) conditions, for adhesion to Caco-2 cells, for bile salt hydrolase secretion, for cholesterol-lowering and cellular cholesterol-binding ability, and for growth inhibition of food-borne pathogens. In addition, safety criteria were assessed, including hemolytic activity and susceptibility to antibiotics. The in vivo test on insulin resistance showed that mice receiving the HFD supplemented with *Pediococcus acidilactici* SK (isolated from human feces) or *P. acidilactici* OLS3-1 strain (isolated from olive fruit) exhibited significantly improved insulin resistance compared to HFD-fed mice or to the normal diet (ND)-fed group.

## 1. Introduction

Nowadays, a growing interest in developing novel functional foods containing probiotic microorganisms is witnessed, as they have been associated with intestinal and host homeostasis, and when administered in adequate amounts may alter the gut microbiome and confer a health benefit to the host [1]. Indeed, the gut microbiome is an important contributor to the pathogenesis of obesity and type 2 diabetes [2]. In recent years, with the rising prevalence of obesity and other metabolic diseases, nutrition-based interventions are fundamental components of many therapeutic strategies for chronic disease treatment and prevention. As a result, interest toward the development of novel functional foods has dramatically increased. Previous studies have shown that probiotics may improve glucose intolerance, hyperinsulinemia, hyperglycemia, and pathologies associated with obesity [3,4,5].

Probiotics must be able to tolerate the acidic conditions of the upper gastrointestinal (GI) tract, in order to deliver the health benefits, as well as colonize and proliferate in the gut [6,7]. Health benefits are mediated through interactions with the host and other gut bacteria, affecting the microbial composition locally and/or stimulating the host immune system [8,9].

At the same time, antimicrobial activity against food-borne pathogens is an important aspect of lactic acid bacteria (LAB) probiotic properties. The antimicrobial activity of probiotics against several healthcare-associated pathogens [10,11,12] has been linked to organic acids, bacteriocins, hydrogen peroxide, diacetyl, and other antimicrobial compounds [13,14,15,16].

Although the probiotic sector has been dominated by species belonging to the genera *Lactobacillus*, *Bifidobacterium*, and *Saccharomyces*, there are some interesting inputs in the market and in the scientific literature of *Pediococcus acidalactici* strains. *P. acidilactici* is one of the main species used in (a) pediocin production, (b) fermentation processes as a starter (co-culture) for avoiding contamination, and (c) probiotic supplements for animals and humans [17]. Some strains of *P. acidilactici* have the ability to lower serum cholesterol levels, promote antioxidant status, and enhance nutrient digestibility [18].

It is worth noting that probiotics provide health-promoting effects that are strain-specific rather than species-specific. Therefore, the food and pharmaceutical industry is continuously seeking novel probiotic strains with unique health properties. Hence, the aim of the present study was to evaluate a series of wild-type strains, isolated from various sources, to manage obesity-associated insulin resistance in a short-term high-fat diet (HFD) mouse model. In this vein, a series of tests regarding functional, safety, and technological criteria were applied, in order to overall assess the effectiveness of novel strains isolated from various sources, targeting mainly their ability to manage obesity-associated insulin resistance.

## 2. Materials and Methods

### 2.1. Bacterial Strains and Culture Conditions

#### 2.1.1. Isolation of Lactic Acid Bacteria (LAB) Strains and Culture Conditions

Greek traditional fermented foods (raisins, olive brine, and olive fruits), as well as human stool samples collected by volunteers randomly after signing a consent declaration form, were used as sources of new wild-type LAB strains. All samples were collected between October and December 2018 and stored at −80 °C until analysis. For the isolation of LAB strains, each sample (5 g) was immersed in de Man, Rogosa, and Sharpe (MRS) broth medium (Condalab, Madrid, Spain) and incubated anaerobically at 37 °C for 48–72 h. Following incubation, 1 mL of each sample was transferred into sterile PPS (Peptone Physiological Solution, 0.1% peptone and 0.85% NaCl in deionized water) test tubes, and 10-fold serial dilution series were prepared in sterile quarter-strength Ringer’s solution (VWR International GmbH, Radnor, PA, USA). Using the spread plate method, 1 mL of each appropriate dilution was inoculated onto acidified MRS agar (Condalab, Madrid, Spain) medium and incubated anaerobically at 37 °C for 72 h (Merck Millipore Anaerobic Jar 2.5 L, Oxoid AnaeroGen 2.5 L Sachets, Burlington, MA, USA). Extended incubations up to 120 h did not yield additional colonies. Multiple isolates with typical morphology on agar plates (small, compact, smooth, convex, opaque, and white) were selected from each sample and picked for routine streaking. Gram staining and catalase tests were performed for lactobacilli confirmation. Each strain was preserved at −80 °C in MRS broth/glycerol (70:30).

Wild-type strains were grown in MRS broth under anaerobic conditions at 37 °C for 24 h. After centrifugation (8000× *g*, 4 °C, 20 min), cell pellets were transferred to −80 °C for 20 h and freeze-dried on a BenchTop Pro (SP Scientific, Warminster, PA, USA) freeze-dryer under vacuum (30–35 Pa) at a condenser temperature of −102 °C for 8 h.

#### 2.1.2. Pathogenic Microbial Strains

*Listeria monocytogenes* NCTC 10527 serotype 4b, *Staphylococcus aureus* ATCC 25923, *Clostridium difficile*, *Salmonella enterica* subsp. *enterica* ser. Enteritidis PT4, and *Escherichia coli* ATCC 25922 were grown in Brain Heart Infusion (BHI) broth (Condalab, Madrid, Spain) at 37 °C for 24 h, excluding *C. difficile*, which was incubated at 37 °C for 48 h. All strains were grown under anaerobic conditions.

### 2.2. Bacterial Identification and Classification

#### 2.2.1. Phenotypic Identification

Cell morphology (colony shape, size, margins, elevation, color, texture, form, and pigmentation), Gram staining, catalase/oxidase tests, and API 50 CHL biochemical tests (Bio-Mèrieux, Craponne, France) were used for phenotypic identification.

#### 2.2.2. Genetic Identification of the 16S rRNA Gene

The 16S ribosomal subunit (rRNA) genetic marker was used for taxonomic classification of the isolates. The 16S rRNA gene sequences contain 9 hypervariable regions (V1-V9) that can be used to identify bacteria and are about 1600 base pairs long [19]. The 16S rRNA gene was amplified utilizing the primers pA: 5′-AGAGTTTGATCCTGGCTCAG-3′ and pH: 5′-AAGGAGGTGATCCAGCCGCA-3′ [20]. In each sample, 4 μL of 5 × MyTaq buffer, 2.5 U of MyTaq (BioLine, Taunton, MA, USA), 0.2 μM of each primer, and 30 ng of each DNA sample were added. The following conditions were used for PCR: initial denaturation at 95 °C for 3 min, followed by 35 cycles of 95 °C for 30 s, 52 °C for 20 s, and 72 °C for 30 s. The PCR products were purified from any by-products (free primers, nucleotides, etc.) with paramagnetic beads (Macherey-Nagel, Düren, Germany), at a ratio of 1/0.8 and a final elution volume of 30 µL. The purified PCR products were subjected to Sanger sequencing for the 16S rRNA gene on the ABI3730xl analyzer (ABI, Los Angeles, CA, USA), using the primers pA and pH.

#### 2.2.3. Whole-Genome Sequencing (WGS) and De Novo Assembly of the Isolates

The wild-type strains were cultured in MRS broth anaerobically at 37 °C for 24 h. From each bacterial culture, 600 µL was centrifuged (11,000× *g* for 3 min), the supernatant was removed, and then the cells were resuspended in 750 µL lysis buffer from the DNA Extraction ZymoBIOMICS DNA Miniprep Kit (Zymo Research, Orange, CA, USA). Genomic DNA was extracted using the ZymoBIOMICS DNA Miniprep DNA Extraction Kit, according to the manufacturer’s instructions, in a final elution volume of 50 μL. The concentration of double-stranded DNA molecules for each sample was measured using the Qubit^®^ dsDNA BR Assay Kit (Life Technologies, Carlsbad, CA, USA). Genome sequencing of the isolates was performed using the paired-end sequencing technology with an Illumina MiSeq sequencer and Nextera XT libraries kit (Illumina, San Diego, CA, USA) and with a MinION sequencer and Rapid Sequencing kit (Nanopore, New York, NY, USA). The quality of the reads was determined using FASTQC. De novo assembly of the raw reads was performed using the SPAdes De Novo Assembler with default parameters, selecting the “careful” parameter to minimize mismatches [21]. Assembly metrics were calculated with CheckM [22].

#### 2.2.4. Genome Annotation

Genome annotation was performed using Prokka with standard parameters [23]. WGS-based antimicrobial susceptibility was performed using ResFinder 4.1 [24,25,26]. The threshold for reporting a match between a gene in the ResFinder database and the input genome was set to 90% and the minimum length to 60% [27,28].

### 2.3. In Vitro Functional Properties Associated with Probiotic Characteristics

#### 2.3.1. Ability to Survive in Simulated Gastrointestinal (GI) Conditions

A static protocol was used to study the survival of wild-type strains under conditions simulating passage through the GI tract, as recently described by Nelios et al. (2022) [29].

#### 2.3.2. Bacterial Adhesion to Differentiated Caco-2 Cells

The human colon cancer cell line Caco-2 was obtained from ATCC (Manassas, Virginia, USA) and was cultured in Dulbecco’s Modified Eagle’s Medium with 4500 mg/L glucose (DMEM high glucose) (Biosera, Nuaille, France) supplemented with 20% *v*/*v* Fetal Bovine Serum (FBS, Gibco, New York, NY, USA), 2 mM L-glutamine (Biosera), 100 U/mL penicillin (Biosera), and 100 U/mL streptomycin (Biosera) at 37 °C in a humidified atmosphere of 5% CO_2_. For differentiation to a colon epithelial phenotype, 500,000 cells/well were seeded in 24-well tissue culture plates (Corning, New York, NY, USA) and cultured in DMEM high glucose, 2 mM L-glutamine, 100 U/mL penicillin, 100 U/mL streptomycin, and 10% *v*/*v* FBS for 21 days with daily medium changes at 37 °C in a humidified atmosphere of 5% CO_2_. On day 21, the culture medium was replaced with a penicillin/streptomycin-free medium; on day 22, cells were washed twice with DMEM high glucose, and the suspension of bacterial cells was added. Bacterial strains were grown in MRS broth medium anaerobically at 37 °C for 24 h, collected by centrifugation (4000 × *g*, 4 °C, 20 min), rinsed twice with sterile quarter-strength Ringer’s solution, and resuspended in penicillin/streptomycin-free medium to a final cell suspension of 10^8^ cfu/mL. Subsequently, 500 μL of bacterial cell suspension was added to each well followed by incubation at 37 °C in a humidified atmosphere of 5% CO_2_ for 2 h. The supernatant from each well was removed, followed by two washes with phosphate-buffered saline (PBS Invitrogen, Waltham, MA, USA), the addition of 500 μL of sterile quarter-strength Ringer’s solution, and detachment of the monolayer using a scraper. The cell concentration (cfu/mL) was determined by serial decimal dilutions in sterile quarter-strength Ringer’s solution; 100 μL of each dilution was plated on MRS agar incubated anaerobically at 37 °C for 72 h, as described above. The attachment potential of wild-type strains was calculated using the following equation [6]:

$$Adhesion\;potential\;(\%)=\frac{cell\;concentration\;(cfu/mL)\;after\;the\;effect\;and\;wash}{cell\;concentration\;(cfu/mL)\;upon\;addition\;of\;bacterial\;cells\;to\;the\;Caco-2\;cell\;monolayer}$$

#### 2.3.3. Bile Salt Hydrolase (BSH) Secretion

In order to determine BSH secretion, sterile paper disks were immersed in 50 μL of cultures of each strain after incubation (cell concentration 10^8^ cfu/mL) and placed on the surface of MRS agar medium enriched with 5 g/L sodium salt of taurodeoxycholic acid (Sigma Aldrich, St. Louis, MO, USA) and 0.37 g/L CaCl_2_. Plates were incubated at 37 °C for 72 h, and the precipitation zone was determined, indicating taurodeoxycholic salt hydrolysis [30,31].

#### 2.3.4. Cholesterol-Lowering Ability

The ability of wild-type strains to reduce cholesterol levels was determined, according to the experimental procedure of Wang et al. (2014) with slight modifications [32]. Briefly, the water-soluble chemical analog of cholesterol ester polyoxyethylene cholesteryl sebacate was used, with a final concentration of 150 μg/mL added to the MRS broth medium. After inoculating the nutrient medium with each strain (postinoculation levels of 10^6^ cfu/mL), the medium was incubated anaerobically at 37 °C for 24 h. The medium was then centrifuged (12,000× *g*, 4 °C, 10 min) (K241R Medium Prime Centrifuge, Centurion Scientific, Chichester, UK) and 100 μL of KOH (33% *w*/*v*) and 200 μL of ethanol (Sigma-Aldrich, Darmstadt, Germany) were added to 100 μL of supernatant and vortexed for 1 min. The mixture was then heated for 15 min at 37 °C, followed by incubation at room temperature for 10 min. Afterward, 200 μL of distilled water and 300 μL of hexane (Sigma-Aldrich, Germany) were added, followed by 1 min of vortexing. The hexane layer was transferred to test tubes and allowed to evaporate before adding 200 μL of 5 mg/mL o-phthalaldehyde (Apollo Scientific, Stockport, UK)–acetic acid (Merck, Darmstadt, Germany) solution and 50 μL of concentrated H_2_SO_4_ (Merck) and vortexing for 2 min. The optical density was measured at 570 nm after a ten-minute period at room temperature (Tecan’s Sunrise, Tecan Trading AG, Männedorf, Switzerland). The cholesterol concentration after incubation was determined using a standard curve (R^2^ = 0.99) and MRS broth-enriched medium with varying cholesterol concentrations. The reduction in cholesterol levels was calculated using the following formula [33]:

$$Cholesterol\;reduction\;(\%)=\frac{(initial\;cholesterol\;concentration-final\;cholesterol\;concentration)\times 100}{initial\;cholesterol\;concentration}$$

#### 2.3.5. Evaluation of Cholesterol Binding by Bacterial Strains

The binding capacity of cholesterol by non-proliferative cells was tested by employing flow cytometry, according to Bosch et al. (2014) with slight modifications [30]. A bacterial culture of 10^9^ cfu/mL was diluted 1:10 in MRS broth medium; 100 μL of this suspension was mixed with the cholesterol chemical analog CholEsteryl BODIPYTM FL C12 (Invitrogen, MA, USA) at a final concentration of 5 mg/mL and incubated at 37 °C for 15 h in the dark with orbital agitation. Cells were collected by centrifugation (3200× *g*, 10 min), washed twice in PBS, and resuspended in PBS at a final volume of 500 μL. Bacterial analysis was conducted using a flow cytometer (Attune NxT flow cytometer, Thermo Fisher Scientific, Waltham, MA, USA) and data analysis was carried out using FlowJo V10 software. Propidium iodide staining (10^−3^ mg/mL, Invitrogen) was used to label nonviable cells. 

#### 2.3.6. Growth Inhibition Activity against Food-Borne Pathogens

Wild-type cultures were grown in MRS broth anaerobically at 37 °C for 24 h, centrifuged (4000 × *g*, 4 °C, 30 min), and filter-sterilized using a 0.22 μm membrane filter (Merck) to obtain cell-free supernatants (CFSs). The pH of the CFSs was determined by a WTW pH 330i device (WTW, Weilheim, Germany).

CFSs growth inhibition activity of non-neutralized and neutralized (pH adjusted to 7.0 by 5M NaOH) CFSs at concentrations of 50 and 25% diluted in MRS broth was investigated, as recently described [29]. Briefly, 100 µL of bacterial culture (10^5^ cfu/mL) and 100 µL of CFS diluted in MRS broth were added in each well of a 96-well microplate to achieve a total volume of 200 µL, followed by anaerobic incubation at 37 °C for 24 h, or 48 h in the case of *C. difficile*. Wells with bacterial inoculum with MRS broth and no CFSs were used as growth controls. Pathogen growth was assessed by measuring optical density (OD) at 620 nm (Tecan’s Sunrise, Τecan Trading AG, Männedorf, Switzerland). The growth inhibition percentage was determined using the following equation [10]:

$$I(\%)=[\frac{\left(OD\;control-OD\;sample\right)}{OD\;control}]\times 100,$$

where I (%) represents the percentage of pathogen growth inhibition in the presence of wild-type strain growth supernatant, OD control is the optical density of pathogen growth in MRS-BHI medium (1:1), and OD sample is the optical density of pathogen growth in the presence of supernatant.

### 2.4. Safety Criteria Assays

#### 2.4.1. Hemolytic Activity

Columbia agar plates (Condalab) containing 5% (*w*/*v*) sheep blood were used to test the strains’ hemolytic activity. Wild-type cultures were grown in MRS broth anaerobically at 37 °C for 24 h and then streaked on Columbia blood agar plates incubated at 30 °C for 48 h. After incubation, the hemolytic activity was evaluated and classified based on red blood cell lysis around the colonies. Green zones were classified as α-hemolysis, clear zones as β-hemolysis, and no zones as γ-hemolysis. Only strains with γ-hemolysis were considered as safe [34].

#### 2.4.2. Susceptibility to Antibiotics

##### Acquired Antimicrobial Resistance Genes

Using the ResFinder 4.1 tool [35,36], the samples were screened for acquired antimicrobial resistance genes. ResFinder is based on a database of more than 2000 resistance genes covering 12 types of antimicrobial resistance agents (aminoglycoside, betalactamase, fluoroquinolone, fosfomycin, fusidic acid, glycopeptide, macrolide-lincosamide-streptograminB, phenicol, rifampicin, sulphoamide, tetracycline, and trimethophorim), which is searched using BLAST.

##### Disk Diffusion Assay

The susceptibility/resistance of wild-type strains to antibiotics was investigated using the disk diffusion assay. The antibiotics (HiMedia Laboratories, Mumbai, India) studied were gentamycin (10 μg/disk), kanamycin (30 μg), streptomycin (10 μg), tetracycline (30 μg), erythromycin (15 μg), clindamycin (2 μg), chloramphenicol (30 μg), ampicillin (10 μg), and vancomycin (30 μg). The method involved inoculating 4 mL of sterile agar at 45 °C with 200 μL of each culture separately, followed by coating the mixture on MRS agar Petri dishes. After 15 min, the antibiotic disks were placed on the surface, followed by incubation at 37 °C for 24 h and determination of inhibition bands around the disks [37]. The results were interpreted according to the cut-off levels proposed by Charteris et al. (1998), with strains considered resistant if inhibition zone diameters were equal to or smaller than 12 mm for gentamycin and ampicillin, 13 mm for kanamycin, erythromycin, and chloramphenicol, 11 mm for streptomycin, 14 mm for tetracycline and vancomycin, and 8 mm for clindamycin [37].

##### Minimum Inhibitory Concentration Determination

The minimum inhibitory concentration (MIC) of antibiotics was determined according to the International Organization for Standardization’s standard procedure (ISO 10932/IDF 223:2010) [38]. Briefly, a cell suspension of 0.16–0.20 optical density determined at 620 nm was prepared from grown cultures of the wild-type strains. Then, 20 μL of the cell suspension was added to 9.980 μL of sterile LSM (HiMedia Laboratories, Mumbai, India) (consisting of 10% *v*/*v* MRS broth and 90% *v*/*v* Iso-Sensitest (IST) medium) to achieve a cell concentration of 10^4^ cfu/mL. The concentration ranges (mg/mL) of the tested antibiotics were gentamicin (0.5–256), tetracycline (0.125–64), chloramphenicol (0.125–64), ampicillin (0.032–16), kanamycin (2–1024), erythromycin (0.016–8), streptomycin (0.5–256), and clindamycin (0.032–16). For MIC determination, 96-well plates were used and 50 μL of each antibiotic dilution and 50 μL of inoculated LSM medium were added to each well, followed by incubation at 37 °C for 48 h. LSM medium inoculated with each strain separately without antibiotic addition was used as a positive control, while sterile LSM medium without inoculation was used as a negative control. The MIC was defined as the lowest antibiotic concentration suppressing visible isolate growth and the results were compared with the microbiological cut-off values specified by the European Food Safety Authority’s (EFSA) recommendations [39].

### 2.5. In Vivo Study Design

#### 2.5.1. Mice

A total of 36 eight-week-old male and female C57BL/6 mice were kept in the specific pathogen-free animal facility of the School of Medicine, University of Crete in Heraklion, Crete, in a 12 h day/night cycle and 21–23 °C conditions. The mice were randomly separated into 9 groups (n = 3); a group of mice fed a normal diet (ND), a group of mice fed a high-fat diet (HFD), and 7 groups of mice fed with HFD supplemented with a potential probiotic strain. Potential probiotic strains were administered daily at a concentration of 10^9^ cfu. All groups were fed a high-fat diet (HFD; 60% energy from fats, catalog number PF4051/D) or a normal chow diet (ND catalog number 4RF21) (purchased from Mucedola, Settimo Milanese, Italy) for 4 days, according to the protocol for short-term HFD-induced insulin resistance [40,41]. Animals were grouped in the same cage per treatment (pooled animals), as highly suggested [42,43] and in alignment with legislative measures, such as the European Union (EU) Directive on animal care and utilization in research and testing [44]. All procedures were conducted in compliance with the national and EU legislation and approved by the Animal Care Committee of the University of Crete, School of Medicine (Heraklion, Crete, Greece; approval number 24/26.10.2018), and from the Veterinary Department of the Region of Crete (Heraklion, Crete, Greece; approval number 269855/5.11.18). Sample size calculations were performed according the Lamorte’s power calculation model (Boston University IACUC guidelines) and, in order to adhere to the 3R principle, no comparison between the different probiotics was planned to reduce the number of mice tested.

#### 2.5.2. Glucose Tolerance Test (GTT)

For GTT, mice were fasted overnight and then injected intraperitoneally with glucose (1 mg/g). Mice were injected intraperitoneally with sterile 35% dextrose (100 μL dextrose/35 g mice) and glucose measurements were conducted in whole-blood specimens collected at time points: 0, 30, and 60 min. All glucose measurements were conducted using a TRUEresult^®^ Twist meter.

#### 2.5.3. Analysis of Fecal Microbiota

Fecal samples were promptly placed on ice after collection and preserved at −80 °C within one hour following collection and until analysis. Feces (~1000 mg) collected from each animal group (pooled samples) were homogenized with sterilized buffered peptone water 0.1 % (LaB M, Heywood, UK) and were subjected to serial dilutions using quarter-strength Ringer’s solution (LaB M). The following tests on microbiological analysis were performed: (i) total aerobic counts on plate count agar (Condalab) at 30 °C for 72 h, (ii) *Enterobacteriaceae* on Violet Red Bile Glucose agar (LaB M) at 37 °C for 24 h, (iii) coliforms on Violet Red Bile agar (LaB M) at 37 °C for 24 h, (iv) *E. coli* on MacConkey agar (LaB M) at 37 °C for 24 h, (v) staphylococci on Baird Parker (Condalab) at 37 °C for 48 h, (vi) yeasts/molds on malt extract (LaB M) at 30 °C for 72 h, (vi) lactobacilli on acidified MRS agar (Condalab) anaerobically at 37 °C for 72 h, as described above, and (vii) enterococci on Kanamycin Aesculin Azide Agar (Condalab) at 37 °C for 48 h. All incubations were further extended up to 120 h, but no extra colonies were observed. Gram staining and catalase tests were performed for lactobacilli confirmation. The results are presented as a log of mean colony-forming units on solid media culture plates containing 30 to 300 colonies per gram of fecal samples.

### 2.6. Statistical Analysis

All experiments were performed at least in triplicate. Statistical significance of the in vitro findings was assessed using factorial analysis of variance (ANOVA), followed by Duncan’s multiple range test. Statistica (v. 12.0, TIBCO Software Inc., Palo Alto, CA, USA) was used to compute significance (*p* < 0.05), coefficients, and ANOVA tables. GraphPad Version 9 InStatsoftware (GraphPad, San Diego, CA, USA) was used to evaluate the in vivo results, which were analyzed using two-way ANOVA with the Bonferroni multiple comparison post-test (*p* < 0.05). Normality tests for all datasets were performed using the Kolmogorov–Smirnov (KS) test.

## 3. Results and Discussion

### 3.1. Bacterial Isolation and Identification

A comprehensive strain collection was undertaken, encompassing multiple isolates from various sources. However, this study selectively focuses on presenting the outcomes for seven isolates distinguished by notable probiotic potential. Each of these isolates originated from a distinct sample, as detailed in Table 1. Specifically, two strains from human stool samples, one from olive brine, three from olive fruit, and one from raisins were isolated and preliminarily identified using API50CH biochemical tests (BioMé-rieux SA, Marcy-l’Étoile, France). All strains were Gram-positive and catalase/oxidase-negative. The wild-type strains were then subjected to WGS (Table 1). All identified species are included in the European Food Safety Authority’s (EFSA) QPS (Qualified Presumption of Safety) list and are thus considered safe for consumption [45].

### 3.2. Ability to Survive through GI Tract Conditions

The reduction in cell viability during passage through the GI tract is a major challenge for probiotic microorganisms. The decrease in cell viability is caused by the low pH of the stomach, proteolytic enzyme activity, and the small intestine’s secretion of bile salts. Tolerance to acidic conditions and bile salts is strain-specific [46,47]. A static in vitro protocol was used in our study to assess the ability of seven wild-type strains to survive under food digestion conditions, focusing on sequential exposure of the strains to simulated oral, gastric, and intestinal conditions.

According to our results (Table 2), exposure to the simulated oral phase had no effect on cell viability, while incubation in the simulated gastric and intestinal phase resulted in a significant (*p* < 0.05) decrease. After simulating the gastric phase, the survival rate ranged from 63.28% to 76.11%. Then, the intestinal phase followed, where the survival rate ranged from 52.14% to 76.91%. After the end of the gastric and intestinal phases, survival rates > 70% were observed for *P. acidilactici* PE11, SK, and OLS2-1 strains (76.91, 71.83, and 70.19%, respectively).

These findings are consistent with previous studies evaluating cell survival of probiotic strains under acidic conditions and in the presence of bile salts; the decrease in viability is most likely due to low pH [46,48]. Notably, when extracellular pH values are low, a similar reduction occurs in the cytoplasmic pH, resulting in the degradation of important enzymes affecting cell membrane fluidity, leading to cell membrane rupture and lysis [47]. It also appears that bacteria with high levels of long-chain unsaturated fatty acids in the cytoplasmic membrane have a better chance of survival in acidic conditions than bacteria with high levels of short-chain saturated fatty acids, as in the latter, membrane fluidity is reduced through hydrophobic interactions [47].

### 3.3. Bacterial Adhesion to Differentiated Caco-2 Cells

The ability of bacteria to adhere to surfaces that mimic the intestinal mucosa has been an important criterion for the selection of probiotic strains, as prolonged cell attachment and eventual colonization of the intestinal epithelium are desirable characteristics of probiotics [49,50]. Furthermore, the adherence of probiotic strains to the colon seems to be associated with antimicrobial activity against pathogens and regulation of the immune system [51]. Therefore, the ability of the isolated strains to adhere to differentiated Caco-2 cells was studied (Table 2), as it is considered strain-specific [50,52]. Adherence percentage ranged between 6.83 and 21.42%. *P. acidilactici* OLS3-1 exhibited the highest adherence capacity (21.42%) (*p* < 0.05), followed by *Lb. pentosus* PE11 and *P. acidilactici* OLL1-1 (adherence capacity 16.80% and 16.75%, respectively). Similar studies evaluating probiotic strains reported that the adherence ability of the genus *Lactobacillus* ranges between 2.0 and 70% [6,53,54], while for the genus *Pediococcus,* between 4.9 and 16.3% [50]. In particular, for *Lb. plantarum* and *Lb. pentosus* species, an attachment capacity of 30–70% has been previously reported [6].

### 3.4. BSH Secretion

The ability to deconjugate bile salt is considered a desirable criterion for selecting potential probiotics as cholesterol-lowering agents [55]. Bacteria with BSH activity promote cholesterol assimilation by deconjugating bile salt, releasing glycine and taurine from its primary form, and producing non-soluble bile salts that can be excreted easily through feces [56,57]. Most probiotics, including species of *Lactobacillus*, have shown tolerance and ability to hydrolyze bile salts [58,59]. However, this activity was proposed to be strain-specific [60,61]. Thus, the isolated wild-type strains were evaluated for BSH secretion by inoculating disks in MRS agar enriched with taurodeoxycholic acid salt (Table 2). The bile acid precipitation zone ranged from 9.0 to 18.0 mm. The highest value (18.0 mm) was observed in *P. acidilactici* OLS3-1 strain, while a precipitation zone of 17.0 mm was observed in *P. acidilactici* SK. The smallest zone (9.0 mm) was recorded in *Lb. plantarum* RS1. Similar results were reported in a study evaluating wild-type strains for bile salt hydrolysis, with a bile acid precipitation zone ranging from 6.0 to 18.0 mm [62].

### 3.5. Reduction of Cholesterol Levels

#### 3.5.1. Cholesterol-Lowering Ability

Several possible mechanisms by which probiotics reduce cholesterol have been proposed, including their ability to deconjugate bile salts using BSH, coprecipitation of deconjugated bile with cholesterol (to be excreted via feces), conversion of cholesterol to coprostanol, binding of LAB cells to cholesterol, and cholesterol incorporation into their cell membrane [63].

A photometric method was applied to evaluate the in vitro ability of wild-type strains to reduce cholesterol levels (Table 2), using the water-soluble chemical analog of cholesterol ester polyoxyethylene cholesteryl sebacate [32]. The wild-type strains reduced cholesterol levels by a percentage that ranged between 15.88 and 43.57%. The greatest reduction (*p* < 0.05) was recorded by *Lb. pentosus* PE11 strain (43.57%). Similar results referring to *Lb. pentosus* species (up to 49%) have been reported [64]. Regarding *P. acidilactici* species, varying data are available, reporting 20% [62], 38% [65], or 68% reduction [50]. As for *Lb. plantarum* species, similar rates of cholesterol reduction (21%) have also been recorded [66].

#### 3.5.2. Evaluation of Cholesterol-Binding Ability of Bacterial Strains

**Figure 1:**
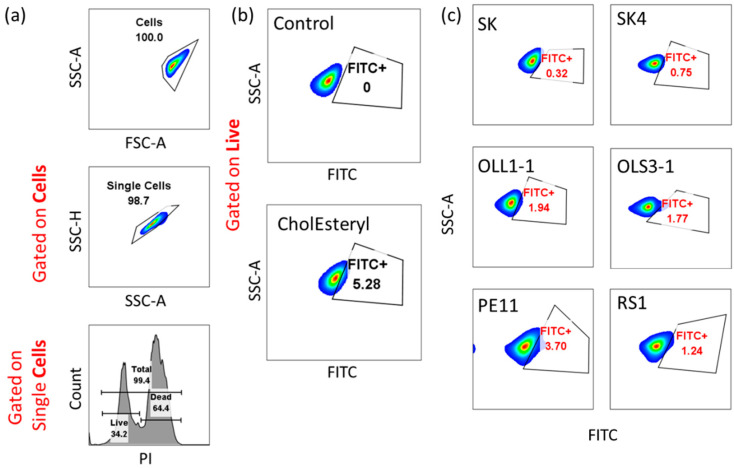
Analysis of cholesterol binding by the different bacterial strains. Binding of cholesterol was analyzed by flow cytometry using the green fluorescent CholEsteryl BODIPYTM FL C12. Bacteria were treated with CholEsteryl for 15 h. Only PI-negative, live cells were analyzed. Positive cells for CholEsteryl (FITC+) were evaluated based on untreated controls analyzed for each strain. (**a**) Representative flow cytometry graphs showing the gating strategy. (**b**) Representative flow cytometry density plots of untreated control bacteria (Control) and bacteria treated with CholEsteryl (CholEsteryl). (**c**) Representative density plots of at least two individual experiments illustrating the cholesterol-binding capacity for the different bacterial strains analyzed. Data were analyzed with FlowJo (v.10, Ashland, OR, USA).

The ability of cholesterol to bind to the cytoplasmic membrane of LAB, which was evaluated using flow cytometry, could affect cholesterol levels. Figure 1 indicates representative flow cytometry graphs and Table 2 shows the percentage of cellular cholesterol binding for the strains analyzed. Cholesterol binding on live cells ranged between 0% (n.d.) in *P. acidilactici* OLS2-1 and 3.61% in *P. acidilactici* OLL1-1.

Cholesterol homeostasis at systemic levels is regulated by intestinal absorption and biosynthesis [67,68,69]. The exact mechanism is currently unknown, but several hypotheses have been formed. Hyperinsulinemia associated with insulin resistance increases fatty acid synthesis and enhances the production of VLDL particles through the upregulation of SREBP1c. However, it is not known whether increased lipogenesis could activate sterol regulatory element-binding protein 2 (SREBP2), which is necessary for cholesterol biosynthesis. In SR-BI transgenic (Tg) mice, cholesterol absorption was inversely correlated with biliary cholesterol concentration, which was found to be increased in obese individuals. In the bloodstream, cholesterol is transported as various lipoproteins, such as VLDL, LDLs, and HDL that can be taken up by scavenger receptors found in the liver and extra-hepatic tissues. Cholesterol can also be acquired through NPC1L1, expressed in the intestine. Many studies have linked insulin resistance to increased cholesterol synthesis and decreased cholesterol absorption; cholesterol is able to saturate intestinal micelles, resulting in poor absorption of dietary sterols. Excretion of cholesterol into bile enlarges the intestinal cholesterol pool and dilutes the dietary cholesterol absorption concentration [69].

### 3.6. Growth Inhibition Activity against Food-Borne Pathogens

Long-term antibiotic use has been linked to changes in the composition of the intestinal microbiota and favors colonization of the intestinal mucosa by pathogenic strains, such as *C. difficile*, *E. coli*, *Staphylococcus aureus*, or *Enterococcus* spp. that are resistant to vancomycin, methicillin, and other antibiotics [70]. Furthermore, toxin-producing *C. difficile* strains are frequently responsible for symptoms of diarrhea after taking antibiotics, as well as colitis [71]. Similarly, *L. monocytogenes* and *Salmonella enterica* strains are often the pathogens responsible for food-borne infections [72]. Colonization of the intestinal mucosa by pathogen-inhibiting microorganisms may provide protection against infections [50]. Competition for nutrients and binding sites on the intestinal mucosa, production of organic acids and antimicrobial substances, and immune system stimulation are some possible mechanisms of action for probiotic strains against pathogens [51]. In this vein, we tested the inhibition activity of seven wild-type strains against common food-borne pathogens.

The percentage of inhibition of *C. difficile* cell growth in the presence of untreated CFSs (Figure S1, Supplementary Materials) ranged from 85 to 97% and 37 to 63% at 50% and 25% concentrations. The percentage of growth inhibition decreased (*p* < 0.05) after pH neutralization of the CFSs (Figure S2, Supplementary Materials), ranging from 4 to 20% and 1 to 18% at 50% and 25% concentrations, respectively.

Regarding the growth inhibition rate of *S.* Enteritidis cells, all untreated CFSs of the studied strains resulted in >93% inhibition (Figure S1) at 50% concentration, while at 25% concentration, a similar growth inhibition was recorded. The percentage of growth inhibition decreased (*p* < 0.05) after CFS neutralization (Figure S2), ranging from 1 to 28% and 6 to 25% at 50% and 25% concentrations, respectively.

Incubation of *E. coli* cells in the presence of untreated CFSs resulted in high inhibition rates (>94%) at 50% CFSs of all strains studied, while at 25%, the inhibition capacity ranged from 52 to 99%. Growth inhibition decreased (*p* < 0.05) after neutralization of CFSs (Figure S2), ranging from 6 to 38% and 4 to 29% at 50% and 25%, respectively.

Regarding the growth inhibition rate of *L. monocytogenes* cells, untreated CFSs caused high growth inhibition rates (>93%) at 50%, while at 25% growth inhibition, they ranged from 52 to 98%, with the exception of *P. acidilactici* OLS3-1 CFS, which caused 38% inhibition (Figure S1). Growth inhibition decreased (*p* < 0.05) after neutralization (13 to 41% and 9 to 31% at 50% and 25%, respectively).

Likewise, untreated CFSs inhibited the growth of *S. aureus* cells by more than 95% at 50% concentration (Figure S1). Similarly high values (>94%) were observed at 25% concentration. The percentage of growth inhibition decreased (*p* < 0.05) after neutralization of the CFSs (Figure S2) down to 4 to 34% and 3 to 31% at 50% and 25%, respectively.

These findings are consistent with previous studies on the inhibitory activity of *Lactobacillus* bacteria supernatant cultures against pathogenic *E. coli*, *L. monocytogenes*, *S.* Enteritidis, and *S. aureus* cells [70,72,73]. Cell growth inhibition rates of *E. coli*, *L. monocytogenes*, and *S.* Enteritidis cells have been recorded in the range of 70 to 93%, 50 to 90%, and 60 to 96%, respectively [73], while after neutralization of supernatants at pH 6.5, a decrease in inhibitory activity against all pathogens was observed [72,73].

The antimicrobial activity against pathogenic cells may be due to the production of organic acids, mainly lactic and acetic acid, which is manifested in the undisturbed form of the acids, as the passage of the molecules across the cytoplasmic membrane is favored [10,73,74], resulting in acidification of the cytoplasm and eventually cell lysis due to rupture of the pathogens’ cytoplasmic membrane [71,73]. The above mechanism explains why pathogen growth is less inhibited during supernatant neutralization.

### 3.7. Safety Criteria

The risk of infection should be assessed when introducing live microorganisms into the diet, and the conclusion that “probiotics are safe” should not be taken for granted. Some European (EU Novel Food Regulation, QPS, and PROSAFE), US (FDA and WHO), and Canadian (Health Canada: NHPR) initiatives have been dedicated to establishing criteria for the safety assessment of probiotics for human use. Records of isolation history, taxonomic identification, and antibiotic resistance genes are all common recommendations [75].

Thus, the hemolytic activity of the seven tested isolates was tested on blood agar plates. None of the examined isolates showed α-hemolytic or β-hemolytic activity when cultivated on Columbia blood agar plates. Instead, the observed hemolytic activity was predominantly γ, signifying a lack of hemolysis or negative hemolytic activity.

#### Susceptibility to Antibiotics

No resistance was observed against the antibiotics tested in silico by the ResFinder 4.1 tool to strains *P. acidilactici* SK, OLL1-1, OLS2-1, OLS3-1, and *Lb. pentosus* PE11, whereas strains *Lb. plantarum* SK4 and RS1 were found to carry the ClpL (ClpL_CP023753) gene (Table 1), which is associated with antibiotic resistance. ClpL, a member of the HSP100 family, is widely distributed in bacteria and plays a key role in survival during stress. This gene has been identified as an important predictor of heat resistance, long-term survival, and antibiotic resistance [76,77]. ClpL is involved in conferring resistance to antibiotics that specifically target the biosynthesis of the cell wall [78]. In the study of Tran et al. (2011), it is showed that a major heat shock protein ClpL/HSP100 could modulate the expression of a cell wall synthesis enzyme PBP2x and subsequently increase cell wall thickness and penicillin tolerance in *Streptococus pneumonia* [79]. Penicillin susceptibility was increased and cell wall thickness was reduced in mutants lacking ClpL compared to the parental strains. On the contrary, a strain overexpressing ClpL exhibited enhanced resistance to penicillin and had thicker cell walls [79]. In another study, when the ATP-binding subunit ClpL (LBP_cg2905), a highly expressed ATP-dependent Clp protease, was deactivated in *Lb. plantarum* 1600 g, there was a noticeable reversal of the ampicillin-resistant phenotype. This indicates that the ClpL protein plays a crucial role in safeguarding bacterial cells against external stress caused by ampicillin [80].

The wild-type strains were then subjected to antibiotic susceptibility testing using the disk diffusion assay (Table S1, Supplementary Materials). All strains showed resistance to vancomycin, kanamycin, and gentamycin and sensitivity in the presence of ampicillin and clindamycin, in agreement with similar studies [6,81].

To confirm the results of antibiotic resistance observed by the disk diffusion assay, the minimum inhibitory concentration (MIC) of antibiotics against cell growth of selected strains (*P. acidilactici* OLS3-1 and *Lb. pentosus* PE11) was determined according to EFSA recommendations (Table S2, Supplementary Materials). These strains were chosen due to their high adherence to Caco-2 cells and reduction of cholesterol levels. According to MIC values, *P. acidilactici* OLS3-1 is considered resistant to kanamycin, gentamycin, tetracycline, and chloramphenicol, while *Lb. pentosus* PE11 is considered resistant to clindamycin and ampicillin. Resistance to kanamycin, gentamycin, and streptomycin was innate [81]. Regarding *P. acidilactici* OLS3-1, the MIC of kanamycin was 128 μg/mL, while similar MIC values have been determined in *P. acidilactici* strains isolated from wine [82], fermented cereal beverages [50], or in strains of human origin [83]. Regarding tetracycline resistance, 12 resistance genes have been reported [83]. However, a recent study evaluating *Pediococcus* strains isolated from cheese or whey has suggested that tetracycline resistance may be innate in this genus [84]. The same study concludes that acquired antibiotic resistance, which occurs through plasmid transfer, results in much higher MIC values than those set by the EFSA, and in these cases, it is only possible to detect antibiotic resistance genes by PCR. At the same time, the heterogeneity of the genus *Pediococcus* and the need to revise the resistance threshold values are highlighted [84]. In addition, in a study determining the MIC of antibiotics in 182 *Lactobacillus* strains, a correlation between species and antibiotic resistance showed that 20% of the strains were resistant to clindamycin [81]. The MIC value of ampicillin and clindamycin in *Lb. pentosus* PE11 was higher than the threshold value (determined at 8 μg/mL), while identical values were reported in an *Lb. pentosus* strain isolated from fermented alcoholic millet beverage [85]. It is worth noting that the resistance of strains to antibiotics may be due to the formation of biofilms, as after the biofilm structure is formed, the properties of the cells are altered compared to those of planktonic cells [86]. Several studies reported the ability of *Lb. pentosus* cells to form biomembranes, especially from strains isolated from olives (such as *Lb. pentosus* PE11), as biomembrane formation is essential for cell survival and olive fermentation [87,88,89].

Safety concerns of newly isolated wild-type bacteria are the first step in evaluating their presumptive use as food additives or supplements [90], as resistance genes localized in plasmids or transposable genetic loci have a high risk of transfer to the normal human microbiota or to potentially pathogenic microbes colonizing the organism, resulting in the horizontal spread of antibiotic resistance genes [39,50]. Both *Pediococcus acidilactici* and *Lactiplantibacillus pentosus* (formerly known as *Lactobacillus pentosus*) are included in the EFSA-QPS list [91] and thus are generally considered safe for consumption. In our study, though, the preliminary antibiotic resistance test was applied with a disk diffusion assay, followed by broth microdilution for MIC determination. Of note, according to EFSA’s (2012) published cut-off values for MIC assessment against common antibiotics specified in species level, determination of vancomycin MIC values is not required for *Pediococcus* spp. Concerning gentamycin and kanamycin, the cut-off values are 16 and 64 μg/mL, respectively. In our study, MIC values determined for *P. acidilactici* OLS3-1 were 32 μg/mL for gentamycin and 128 μg/mL for kanamycin, slightly higher (2-fold higher) than the recommended concentration. However, these microbes are unlikely to have acquired antimicrobial resistance genes to clinically relevant antimicrobials, as documented by the Resfinder 4.1 tool, but further research is recommended to prove that resistance genes are not on mobilizable elements, such as plasmids or transposons.

### 3.8. In Vivo Assays

#### 3.8.1. Effect of Probiotic Strains on Glucose Intolerance and Insulin Resistance

Following in vitro screening assays for their potential probiotic properties (Table 2), wild-type strains were further evaluated in vivo for their capacity to confer health benefits to the host. Therefore, a model of HFD-induced insulin resistance was used, where mice received HFD for a short period to develop insulin resistance without increases in body weight, therefore allowing us to dissect the effect on insulin resistance rather than more complex changes that occur in obesity. Adequate concentrations of freeze-dried cells were administered to mice daily.

**Figure 2:**
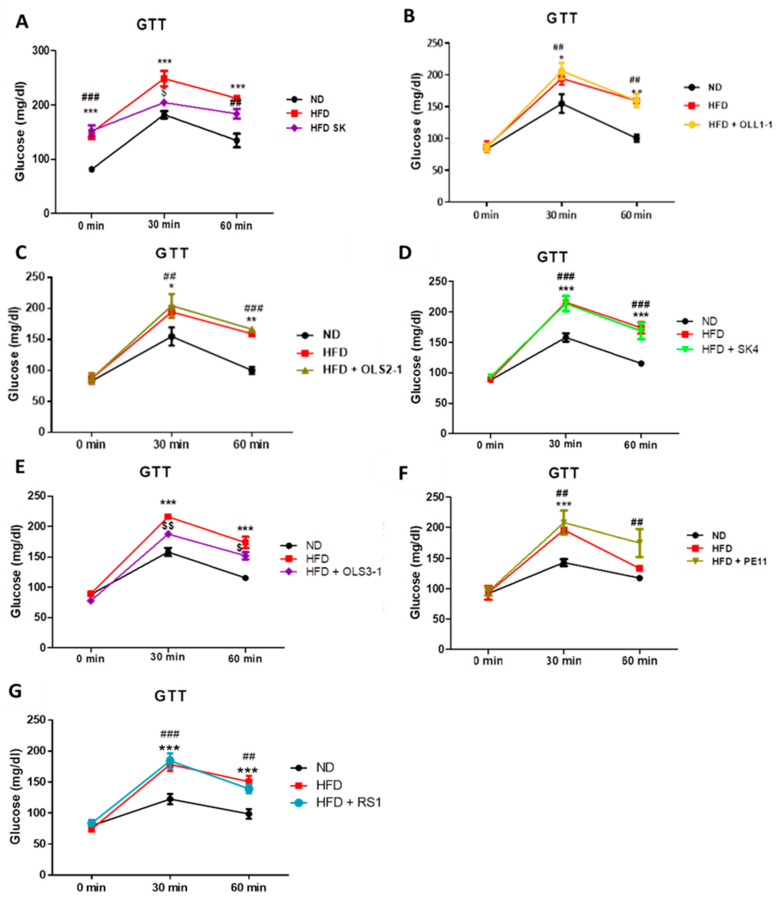
Glucose tolerance test in mice that received (**A**) normal diet, ND, high-fat diet, HFD, and HFD supplemented with *P. acidilactici* SK, (**B**) ND, HFD, and HFD supplemented with *P. acidilactici* OLL1-1, (**C**) ND, HFD, and HFD supplemented with *P. acidilactici* OLS2-1, (**D**) ND, HFD, and HFD supplemented with *Lb. plantarum* SK4, (**E**) ND, HFD, and HFD supplemented with *P. acidilactici* OLS3-1,(**F**) ND, HFD, and HFD supplemented with *Lb. pentosus* PE11, and (**G**) ND, HFD, and HFD supplemented with *Lb. plantarum* RS1. * *p*< 0.05, ** *p*< 0.01, *** *p*< 0.001, ND versus HFD, ^##^ *p*< 0.01, ^###^ *p*< 0.001, ND versus HFD + potential probiotic, ^$^ *p*< 0.05, ^$$^ *p*< 0.01 HFD versus HFD + potential probiotic.

Short-term HFD feeding resulted in the development of insulin resistance in mice compared to mice fed a chow diet (normal diet, ND), as measured by the glucose tolerance test (Figure 2). In Figure 2, the impact of dietary supplementation of *P. acidilactici* SK, OLL1-1, OLS2-1, *Lb. plantarum* SK4, RS1, and *Lb. pentosus* PE11 on the development of insulin resistance is shown. Mice that received HFD supplemented with *P. acidilactici* SK had significantly improved insulin resistance compared to HFD-fed mice (Figure 2A). On the contrary, administration of *P. acidilactici* OLL1-1, *P. acidilactici* OLS2-1, and *Lb. plantarum* SK4 resulted in increased glucose levels during the test, similar to the HFD-fed group (Figure 2B–D). Mice that received short-term dietary supplementation with the *P. acidilactici* OLS3-1 strain showed increased blood glucose levels during the test compared to ND-fed mice, but better glucose tolerance compared to HFD-fed mice (Figure 2E). Mice fed an HFD supplemented with *Lb. pentosus* PE11 and *Lb. plantarum* RS1 developed insulin resistance and showed no improvement compared to the HFD-fed group (Figure 2F,G).

#### 3.8.2. Preliminary Evaluation of the Effect of Potential Probiotic Supplementation on Fecal Microbiota

**Figure 3:**
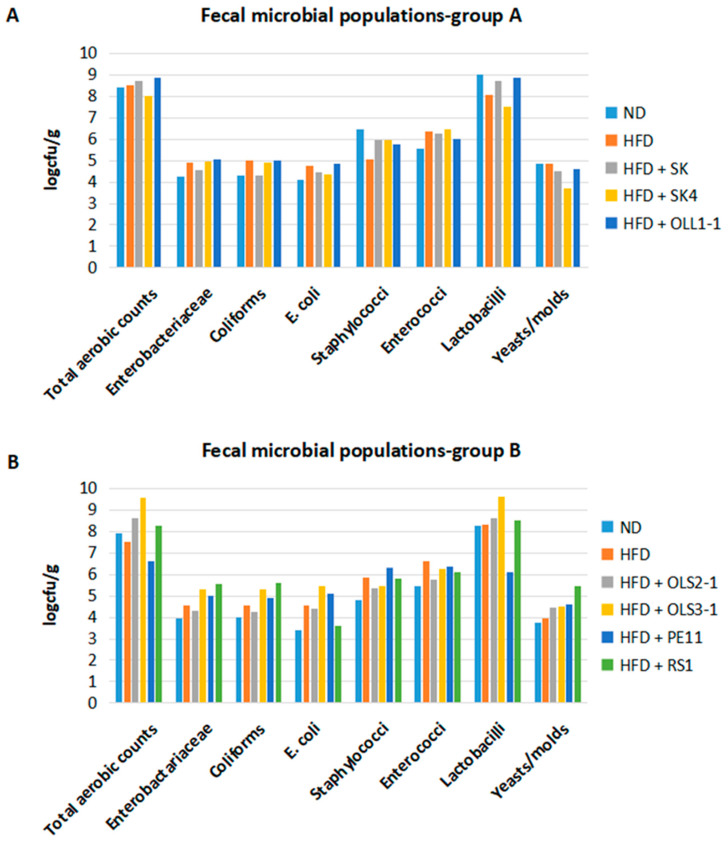
Fecal microbial populations in mice fed (**A**) normal diet, ND, high-fat diet, HFD, and HFD supplemented with *P. acidilactici* SK (HFD + SK), *Lb. plantarum* SK4 (HFD + SK4), and *P. acidilactici* OLL1-1 (HFD + OLL1-1), and (**B**) ND, HFD, and HFD supplemented with *P. acidilactici* OLS2-1 (HFD + OLS2-1), *P. acidilactici* OLS3-1 (HFD + OLS3-1), *Lb. pentosus* PE11 (HFD + PE11), and *Lb. plantarum* RS1 (HFD + RS1).

Disruptions in the gut microbiota have been linked to the development of obesity and type 2 diabetes. Evidence suggests that metabolic disorders result in an increased *Firmicutes* to *Bacteriodetes* ratio and decreased microbial diversity in the fecal microbiota [92,93]. We, therefore, evaluated the effect of short-term supplementation of potential probiotics on fecal microbiota by microbiological analysis in the groups that received an ND, HFD, and HFD supplemented with the different wild-type strains (Figure 3). Since mice were caged per group, analysis of pooled fecal samples was performed, which provided an indication of changes in the gut microbiota composition of each group.

Total aerobic bacteria, lactobacilli, and yeasts/molds did not differ between HFD- and ND-fed mice. Coliforms, *Enterobacteriacae*, enterococci, and *E. coli* were increased in mice fed an HFD compared to ND-fed mice. All probiotic strains administered resulted in changes in the fecal microbiota of mice compared to the HFD group; however, not all of these strains resulted in improved glucose tolerance. Supplementation of diet with *P. acidilactici* SK resulted in increased lactobacilli levels compared to the HFD group. In addition, a reduction in coliforms, *Enterobacteriacae*, and *E. coli* counts was evident in the microbiota of mice fed an HFD supplemented with *P. acidilactici* SK compared to mice fed an HFD alone. Mice fed an HFD supplemented with *P. acidilactici* OLS3-1 showed increased total aerobic counts, and levels of coliforms, *Enterobacteriacae*, *E. coli*, and lactobacilli compared to mice fed with HFD.

Overall, in vivo screening of a series of potential probiotic strains showed that *P. acidilactici* SK isolated from human feces and *P. acidilactici* OLS3-1 isolated from olive fruit had a significant effect in improving glucose tolerance.

Obesity and insulin resistance are associated with a lower abundance of *Bacteroidetes*, since they possess fewer enzymes implicated in the metabolism of lipids and carbohydrates found in the diet. Increased levels of *Lactobacillus* (phylum *Firmicutes*) and *Bifidobacterium* (phylum *Actinobacteria*) species were found in obese compared to lean individuals. However, this increase seems to be species-specific, since higher levels of *Limosilactobacillus reuteri* and lower levels of *Lacticaseibacillus casei/paracasei* and *Lactiplantibacillus plantarum* were associated with obesity and insulin resistance [94]. Increased levels of *Enterococcus* spp. and *Enterobacteriaceae* species are correlated with HFD consumption. In addition, increased levels of *Staphylococcus* and *E. coli* were found in obese individuals and overweight pregnant women [95]. Our results showed that a short-term HFD induces glucose intolerance and insulin resistance and increases the levels of *Enterobacteriacae*, enterococci, and *E. coli* with no effect on lactobacilli. Important changes in the composition of the gut microbiota were found in mice that received an HFD supplemented with *P. acidilactici* SK and *P. acidilactici* OLS3-1 strains.

Probiotic strains used for management of obesity, mainly *Lactobacillus* and *Bifidobacterium* species, result in increased total aerobes, anaerobes, *Lactobacillus*, *Bifidobacteria*, and *Streptococcus* and reduced total coliforms and *E.coli* compared to control subjects [96].

In line with the literature, *P. acidilactici* SK supplementation resulted in increased lactobacilli levels and a reduction in coliforms, *Enterobacteriacae*,and *E. coli* levels. *P. acidilactici* OLS3-1 also led to increased lactobacilli, but also increased levels of other species. Bifidobacteria abundance should also be measured since they are significantly affected during metabolic disturbances. As changes in intestinal microbiota during obesity and insulin resistance are species-specific, a more detailed analysis of the gut microbiome that could provide statistically significant data will reveal the exact microbial species that colonize the intestine upon each probiotic strain administration.

Further evaluation of these potential probiotic strains for their impact on other features of obesity could include their effect on body and adipose tissue weight gain, liver lipid metabolism, and dyslipidemia.

## 4. Conclusions

In conclusion, a series of novel potential probiotic strains, isolated from various sources, was evaluated in vitro for survival in simulated gastrointestinal (GI) conditions, adhesion to Caco-2 cells, bile salt hydrolase secretion, cholesterol-lowering and cellular cholesterol-binding ability, and growth inhibition of food-borne pathogens, along with hemolytic activity and susceptibility to antibiotics. Application of a diet-induced obesity mouse model showed that *P. acidilactici* SK and *P. acidilactici* OLS3-1 strains delayed the development of insulin resistance found early in obesity. However, more research is still required to further reveal potential significant properties that could assist in the prevention of type 2 diabetes.

## Figures and Tables

**Table 1:** Classification and genome characteristics of the novel wild-type isolates.

Isolate Code	SK	SK4	OLL1-1	OLS2-1	OLS3-1	PE11	RS1
**Source of isolation**	Human stool samples	Human stool samples	Olive brine	Olive fruit	Olive fruit	Olive fruit	Raisins
**Classification**	*Pediococcus acidilactici*	*Lactiplantibacillus plantarum* subsp. *plantarum*	*Pediococcus acidilactici*	*Pediococcus acidilactici*	*Pediococcus acidilactici*	*Lactiplantibacillus pentosus*	*Lactiplantibacillus plantarum* subsp. *plantarum*
**Classification method**	Illumina/ Nanopore	Illumina	Illumina/ Nanopore	Illumina	Illumina	Illumina	Illumina
**Number of contigs**	11	45	610	327	390	150	411
**Genome length (bp)**	2,044,391	3,226,900	5,199,416	2,171,522	2,209,033	3,897,459	3,421,158
**N50**	1,082,018	481,816	52,231	394,208	393,990	166,230	156,534
**Completeness**	99.38	99.07	98.27	99.38	99.38	99.38	99.07
**GC content (%)**	42	44	43	41	42	46	44
**Predicted genes**	2030	3044	5875	2070	2124	3574	3439
**CDSs**	1933	3029	5485	1994	1961	3508	3207
**rRNAs**	14	8	7	9	41	26	3
**tRNAs**	56	65	58	63	54	73	57
**tmRNAs**	1	1	2	1	1	1	1
**misc_RNAs**	26	44	0	40	0	52	0
**Antibiotic resistance genes**	No resistance	Resistance gene ClpL	No resistance	No resistance	No resistance	No resistance	Resistance gene ClpL

**Table 2:** Basic characteristics of the novel wild-type isolates.

Isolate Code	Classification	Survival Rate (%) during In Vitro Gastrointestinal Tract Conditions			Percentage (%) Adherence to Caco-2 Cells	Bile Acid Precipitation Zone (mm)	Percentage (%) Reduction of Cholesterol Levels	Cellular Cholesterol Binding (%)
		Simulated Oral Phase	Simulated Gastric Phase	Simulated Intestinal Phase				
**SK**	*Pediococcus acidilactici*	99.69 ± 0.06	71.73 ± 1.12 ^c^	71.83 ± 0.24 ^d^	11.75 ± 2.47 ^ab^	17.0	29.24 ± 3.57 ^cd^	0.39 ± 0.03 ^a^
**SK4**	*Lactiplantibacillus plantarum* subsp. *plantarum*	99.36 ± 0.37	63.97 ± 0.37 ^ab^	62.92 ± 1.34 ^b^	8.59 ± 0.31 ^a^	16.5	21.22 ± 3.22 ^ab^	3.42 ± 0.15 ^ab^
**OLL1-1**	*Pediococcu sacidilactici*	99.27 ± 0.14	63.93 ± 0.12 ^ab^	52.51 ± 0.54 ^a^	16.75 ± 4.26 ^bc^	14.0	15.88 ± 2.15 ^b^	3.61 ± 2.36 ^b^
**OLS2-1**	*Pediococcus acidilactici*	99.68 ± 0.08	76.11 ± 0.41 ^d^	70.19 ± 0.30 ^c^	8.65 ± 0.87 ^a^	16.0	22.36 ± 3.45 ^a^	n.d.
**OLS3-1**	*Pediococcus acidilactici*	99.58 ± 0.24	71.89 ± 0.97 ^c^	64.16 ± 0.21 ^b^	21.42 ± 3.25 ^c^	18.0	32.96 ± 1.84 ^d^	1.58 ± 0.27 ^ab^
**PE11**	*Lactiplantibacillus pentosus*	99.78 ± 0.41	65.41 ± 0.57 ^b^	76.91 ± 0.13 ^e^	16.80 ± 3.31 ^bc^	15.0	43.57 ± 1.55 ^e^	2.46 ± 1.75 ^ab^
**RS1**	*Lactiplantibacillus plantarum* subsp. *plantarum*	99.65 ± 0.18	63.28 ± 0.06 ^a^	52.14 ± 0.91 ^a^	6.83 ± 1.48 ^a^	9.0	25.24 ± 0.47 ^ac^	0.89 ± 0.49 ^ab^

Significant differences (*p* < 0.05) within each column are indicated by superscript letters. n.d.: not detected.

## Data Availability

The data presented in this study are available on request from the corresponding author. The data are not publicly available due to restrictions of the funding authorities. Clostridium difficile was kindly provided by the Laboratory of Clinical Microbiology, Sismanoglio General Hospital, Athens, Greece. Salmonella enterica subsp. enterica ser. Enteritidis PT4 was kindly provided by the Laboratory of Microbiology and Biotechnology, Agricultural University of Athens, Greece. Escherichia coli ATCC 25922 was kindly provided by Dr. Nisiotou A., Athens Wine Institute, ELGO-DIMITRA, Lykovrysi, Greece.

## Supplementary Materials

The following supporting information can be downloaded at: https://www.mdpi.com/article/10.3390/microorganisms12020231/s1, Figure S1: Growth inhibition activity of untreated cell-free supernatants (CFSs) of the novel wild-type strains against food-borne pathogens. U: untreated supernatant.; Figure S2: Growth inhibition activity of neutralized cell-free supernatants (CFSs) of the novel wild-type strains against food-borne pathogens. N: neutralized supernatant.; Table S1: Antibiotic resistance testing of the novel wild-type strains by the disk diffusion assay; Table S2: Minimum inhibitory concentration (MIC) values of antibiotics (μg/mL) against cell growth of selected wild-type strains.
