# Supplementary material for: Novel Wild-Type Pediococcus and Lactiplantibacillus Strains as Probiotic Candidates to Manage Obesity-Associated Insulin Resistance
Source: Microorganisms. 2024 Jan 23;12(2):231. doi: 10.3390/microorganisms12020231 (PMC10891751; doi:10.3390/microorganisms12020231)
Supplement: Supplementary file 1 [file microorganisms-12-00231-s001.zip › microorganisms-2765611-supplementary.pdf]

Supplementary Material

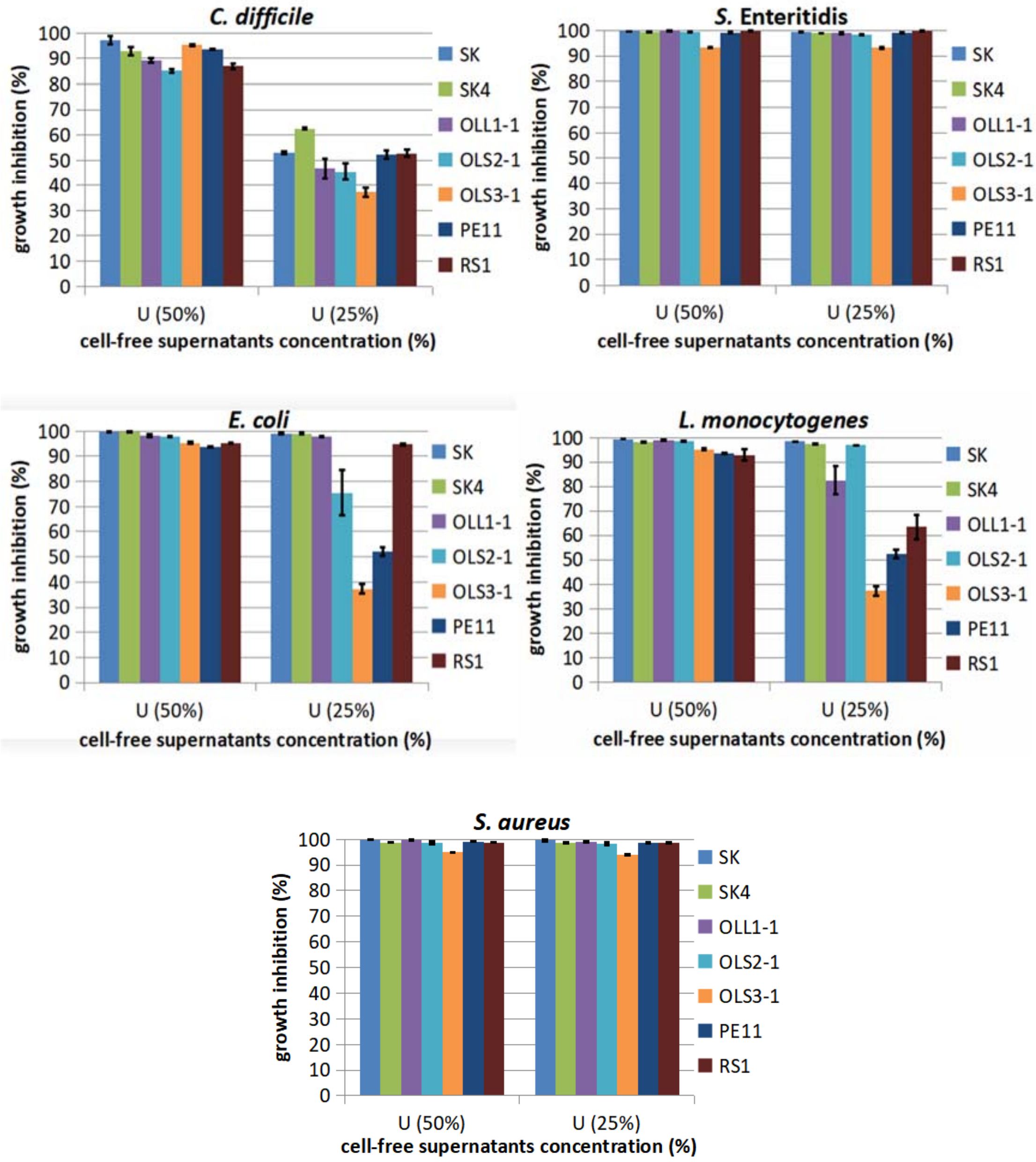

Figure S1. Growth inhibition activity of untreated cell-free supernatants (CFSs) of the novel wild-type strains against food-borne pathogens. U: Untreated supernatant.

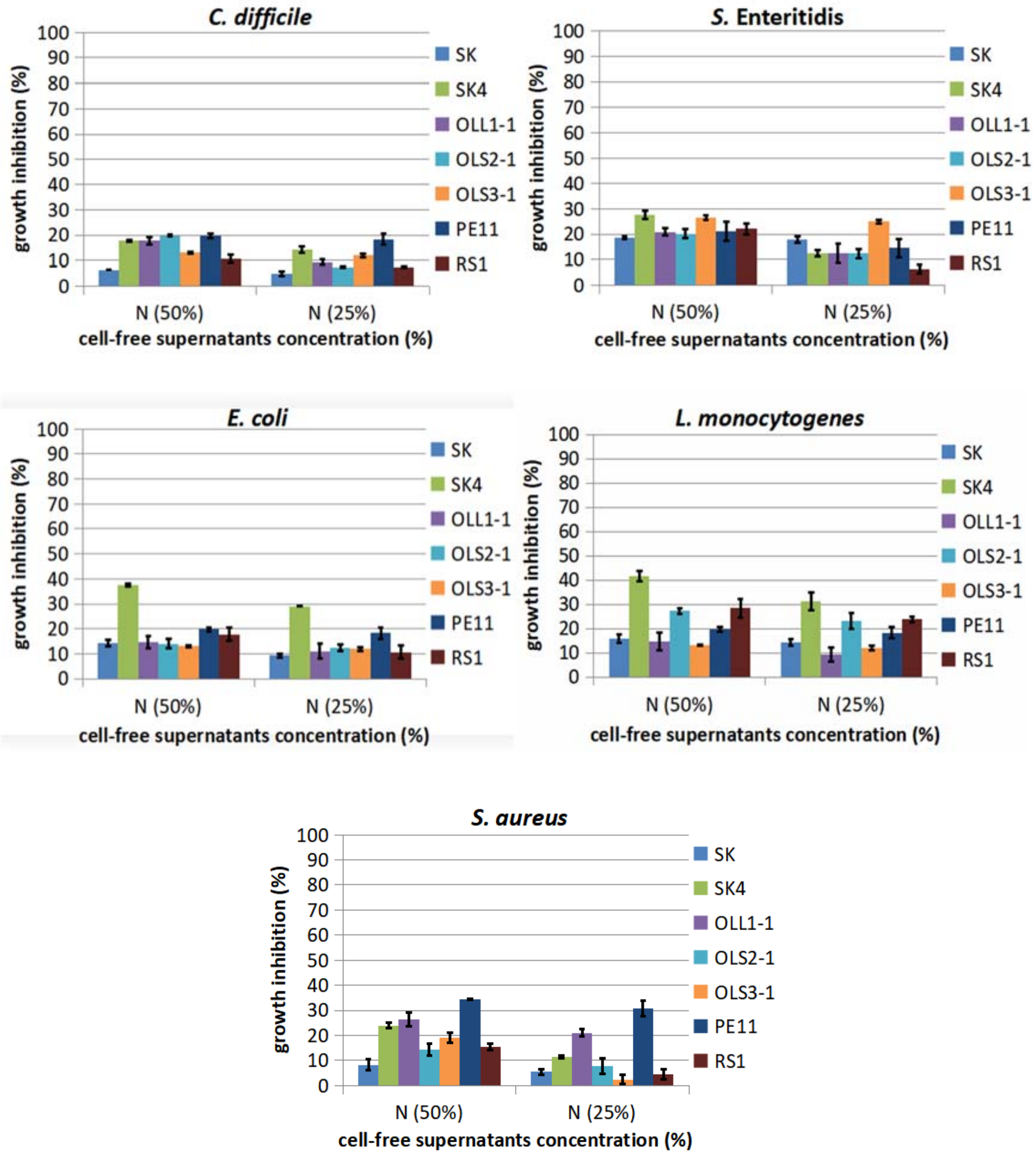

Figure S2. Growth inhibition activity of neutralized cell-free supernatants (CFSs) of the novel wild-type strains against food-borne pathogens. N: Neutralized supernatant.

Table S1. Antibiotic[a] resistance[b] testing of the novel wild-type strains by the disc diffusion assay.

Isolate code	Classification	Antibiotics								
		VA	AM	KA	GM	SM	TE	E	CL	C
SK	*Pediococcus acidilactici*	R	S	R	R	R	R	I	S	I
SK4	*Lactiplantibacillus plantarum* subsp. *plantarum*	R	S	R	R	R	S	S	S	S
OLL1-1	*Pediococcus acidilactici*	R	S	R	R	R	R	S	S	S
OLS2-1	*Pediococcus acidilactici*	R	S	R	R	R	R	I	S	I
OLS3-1	*Pediococcus acidilactici*	R	S	R	R	S	R	I	S	I
PE11	*Lactiplantibacillus pentosus*	R	S	R	R	R	S	S	S	S
RS1	*Lactiplantibacillus plantarum* subsp. *plantarum*	R	S	R	R	R	I	I	S	I

[a] VA: Vancomycin, AM: Ampicillin, KA: Kanamycin, GM: Gentamicin, SM: Streptomycin, TE: Tetracyclin, E: Erythromycin, CL: Clindamycin, C: Chloramphenicol.

[b] S: Sensitive, I: Moderately sensitive or intermediate, R: Resistant.

Table S2. Minimum Inhibitory Concentration (MIC) values of antibiotics[a] (µg/mL) against cell growth of selected wild-type strains.

Isolate code	Classification	Antibiotics							
		**AM**	**KA**	**GM**	**S**	**TE**	**E**	**CL**	**C**
OLS3-1	*Pediococcus acidilactici*	2	128[R]	32[R]	64	32[R]	1	0.5	8[R]
PE11	*Lactiplantibacillus pentosus*	8[R]	32	2	n.r.[1]	32	1	8[R]	8

[a] AM: Ampicillin, KA: Kanamycin, GM: Gentamicin, S: Streptomycin, TE: Tetracyclin, E: Erythromycin, CL: Clindamycin, C: Chloramphenicol.

[R] R: Resistant [38].

[1] n.r.: Not required [38].
